# Metabolism of asparagine in the physiological state and cancer

**DOI:** 10.1186/s12964-024-01540-x

**Published:** 2024-03-06

**Authors:** Qiong Yuan, Liyang Yin, Jun He, Qiting Zeng, Yuxin Liang, Yingying Shen, Xuyu Zu

**Affiliations:** 1 Cancer Research Institute, The First Affiliated Hospital, Hengyang Medical School, University of South China, Hengyang, Hunan 421001 PR China; 2 Department of Clinical Laboratory Medicine, The First Affiliated Hospital, Hengyang Medical School, University of South China, Hengyang, 421001 Hunan China; 3 Department of Spine Surgery, The Nanhua Affiliated Hospital, Hengyang Medical School, University of South China, Hengyang, China

**Keywords:** Asparagine, Asparaginase synthase, Cancer, Metabolism, Stress response

## Abstract

Asparagine, an important amino acid in mammals, is produced in several organs and is widely used for the production of other nutrients such as glucose, proteins, lipids, and nucleotides. Asparagine has also been reported to play a vital role in the development of cancer cells. Although several types of cancer cells can synthesise asparagine alone, their synthesis levels are insufficient to meet their requirements. These cells must rely on the supply of exogenous asparagine, which is why asparagine is considered a semi-essential amino acid. Therefore, nutritional inhibition by targeting asparagine is often considered as an anti-cancer strategy and has shown success in the treatment of leukaemia. However, asparagine limitation alone does not achieve an ideal therapeutic effect because of stress responses that upregulate asparagine synthase (ASNS) to meet the requirements for asparagine in cancer cells. Various cancer cells initiate different reprogramming processes in response to the deficiency of asparagine. Therefore, it is necessary to comprehensively understand the asparagine metabolism in cancers. This review primarily discusses the physiological role of asparagine and the current progress in the field of cancer research.

## Introduction

Amino acids, the basic units of proteins, are widely involved in the formation of energy, synthesis of macromolecules, and signal transduction in cells. They are essential for the survival of cancer cells. Amino acid metabolism is an important metabolism process in cancer cell and has attracted the extensive research attention, particularly the metabolism of non-essential amino acids. Among them, the most studied non-essential amino acid is glutamine that contributes to cancer cell proliferation, invasion, and migration. Glutamine is the highest content of amino acids in plasma, but many cancer cells easily produce glutamine addiction due to the high demands for nutrient, especially in cancer cells that enhanced myelocytomatosis oncogene (MYC) protein expression [1]. Therefore, glutamine metabolism has become important targets for diagnostic imaging and treatment of cancers [2]. With the development of clinical research, investigators have gradually enhanced the study of asparagine to provide a vital theoretical basis for its use as a cancer-treatment target.

Asparagine, a non-essential amino acid, can be produced by de novo synthesis in addition to being obtained from food. Two enzymes are involved in asparagine metabolism: asparagine synthase (ASNS), which catalyses glutamine- or ammonia-dependent asparagine synthesis from aspartate, and asparaginase (ASNase), which hydrolyses asparagine to aspartate. Aspartate is mainly generated in the mitochondria through the respiratory chain [3]. In humans, ASNS is expressed in several organs, and the highest levels of ASNS activity are observed in the pancreas. While ASNase is expressed in only a few human organs, such as the liver and kidneys. Numerous studies indicated that asparagine metabolism is essential for the growth and development of cancer cells [4]. Briefly, asparagine metabolism in cancers mainly refer to cancer cells upregulate ASNS expression and further catalyses synthesis of asparagine via various signaling pathways in order to meet the needs of growth, and the mechanism of asparagine involved in cancer cells growth and metastasis.

Nutritional restrictions are often used against cancer because of the high basal metabolic rate and nutritional requirements of cancer cells [2]. One of the most significant therapeutic strategies is asparagine restrictions. For cancer cells, the amount of asparagine synthesised by themselves cannot meet their need for asparagine; therefore, they are more sensitive to exogenous asparagine than normal cells. Clinically, ASNase has successfully suppressed leukaemia by specifically reducing circulating asparagine levels [5]. However, ASNase is not as effective for treating other solid cancers. Investigators have explored the reasons for the poor efficacy of ASNase. Because asparagine is obtained from circumstance and ASNS-dependent de novo synthesis, different cells show different sensitivities to ASNase owing to different intracellular levels of ASNS expression. While ASNS protein expression levels are closely related to many regulators in the cells. Different cancer cells have unique metabolic characteristics, and they specifically adjust asparagine metabolism to meet their energy and nutrient requirements [6]. Therefore, a comprehensive understanding of the metabolism and role of asparagine has important clinical implications and potential applications [7]. This will help increase the therapeutic efficacy of ASNase during cancer therapy, search for more effective treatment strategies and diagnostic approaches, and reduce the risk of side effects.

### Physiological functions of asparagine

#### The asparagine-dependent metabolism of the nutrients

In proliferating cells, asparagine is one of the least abundant non-essential amino acids [4]; however, it is essential for cell survival. A previous study indicated that the main purpose of mitochondrial respiration was to synthesise asparagine [8]. With the increasing research on asparagine, the role of it is not just limited to as the substrates for protein synthesis. As early as 1883, Schulze and Bosshard discovered a tendency for the spontaneous deamidation of asparagine under mild conditions. However, this process does not require catalytic enzymes. It is primarily determined by the amino acid sequence surrounding asparagine and is governed by multiple layers in the protein interior. When some amino acids are altered, they may cause deamidation of key asparagine molecules around them, making asparagine a regulator of protein turnover [9]. There is a negative relationship between the asparagine content and protein lifetime. However, some studies have suggested that spontaneous deamidation of asparagine generates an isoaspartate residue that hampers protein function and induces disorders associated with senescence [10]. Sequence- and structure-based methods can detect asparagine deamidation in proteins [11]; thus, we can predict the function of proteins through these methods.

Asparagine also plays an important regulatory role in the metabolism of other nutrients. Compared with other amino acids, asparagine can activate the mammalian target of rapamycin complex 1 (mTORC1) through ADP-ribosylation factor 1 (ARF1) in a Rag GTPase-independent manner [12]. mTORC1 phosphorylates ribosomal protein S6 kinase 1 (S6K1) and eukaryotic translation initiation factor 4E (eIF4E)-binding protein 1 (4E-BP1) when stimulated by cell growth signals [13]. S6K1, one of these targets, mediates the phosphorylation of carbamoyl-phosphate synthetase 2, aspartate transcarbamoylase, and dihydroorotatase (CAD) at Ser1859 which catalyses the de novo synthesis of pyrimidine [14]. Moreover, asparagine can directly offer γ-nitrogen for the biosynthesis of purine and pyrimidine [15]. Phosphorylated 4E-BP1, another mTORC1 target, blocks its binding to eIF4E, enabling it to form the eIF4E complex required for initiating protein translation [16].

**Fig. 1:**
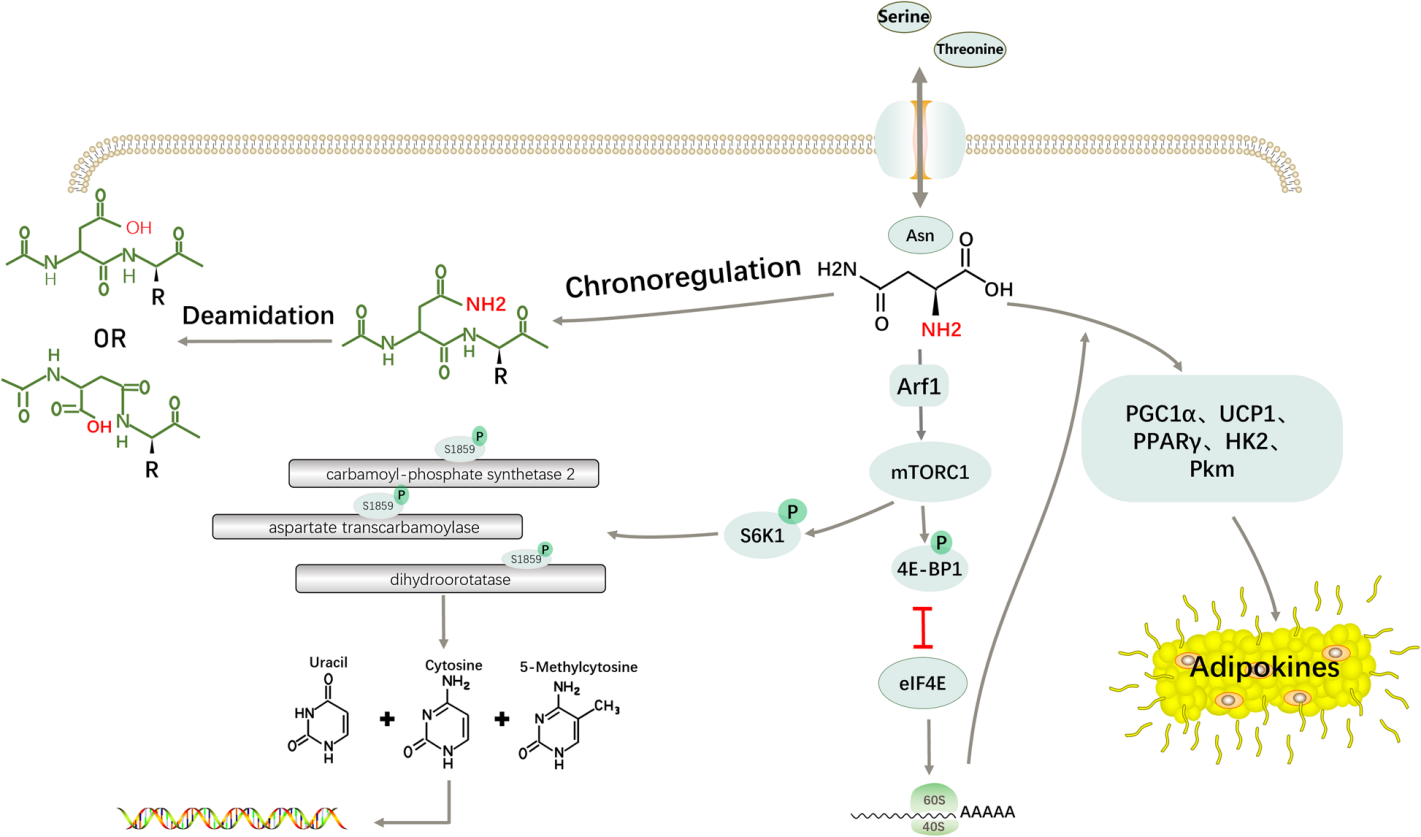
Connections between asparagine and other nutrients. In addition to comprising the basic component of the protein-peptide chains, asparagine regulates the intake of amino acids by serving as an amino acid exchange factor and protein turnover by serving as a regulator. Asparagine also plays a role in the synthesis of nucleic acid molecules, the glycolysis process, and heat production in adipocytes through mTORC1 signalling cascades

The role of asparagine has been preliminary studied in adipose tissue as well. Brown and beige adipocytes primarily consume energy generated by the oxidation of fatty acids and glucose in the form of heat [17]. When brown adipocytes were cultured in a medium containing asparagine, the expression levels of lipogenic and thermogenic genes increased compared to the control group. In acute cold exposure experiments, an improvement in cold resistance was observed in mice after supplementation with asparagine. In contrast, when treated with ASNase, acute cold stimulation induced hypothermia in mice. Further metabolomic analysis and isotope tracing showed that the levels of key enzymes and glycolytic intermediates were significantly increased. It has been proposed that glucose is the primary source of thermogenesis in the adipose tissue [18]. When adipocyte glucose transporters (such as glucose transported type 1 (Glut1), Glut4, hexokinase 2 (HK2), or pyruvate kinase (Pkm)) are knocked down, both thermogenesis and oxygen consumption are reduced in brown adipose tissue (BAT) [19]. Therefore, asparagine promotes adipocyte thermogenesis, at least in part, by increasing glycolysis. In addition, these regulatory mechanisms are involved in the mTORC1 signalling cascade [20] (Fig. 1).

### Asparagine is important during glutamine deprivation

**Fig. 2:**
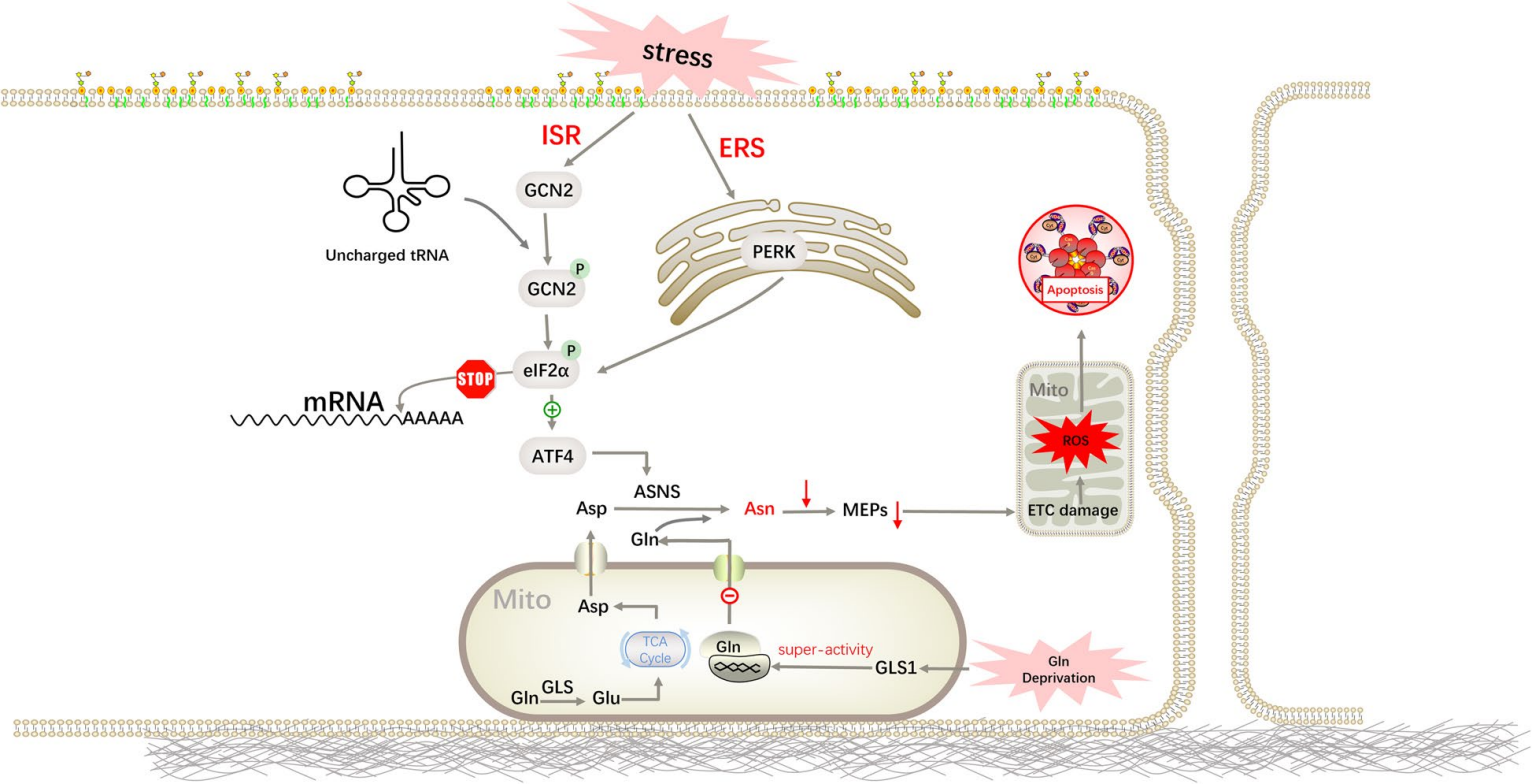
The role of asparagine during cellular stress. Asparagine is synthesized under the catalytic action of ASNS using aspartate and glutamine as raw materials, in which glutamine serves as the carbon source as well as the nitrogen source. When cells are under stress due to the shortage of nutrients, ISR or ERS are initiated, which increases the production of asparagine to maintain cell growth and development by upregulating the expression of ASNS. When raw materials are deficient or the expression of ASNS fails to be activated, cells cannot synthesize sufficient asparagine, leading to apoptosis through ETC damage. Mito, Mitochondrion

In addition to maintain the most basic physiological metabolism, asparagine is particularly crucial when cells are starved for nutrients, especially glutamine. Glutamine is required for de novo asparagine synthesis. It is both a carbon and nitrogen source for asparagine. Glutaminase catalyses the hydrolysis of glutamine to glutamate and ammonia. Glutamate then enters the tricarboxylic acid cycle (TCA cycle) and the respiratory chain to generate aspartate in the mitochondria, which subsequently enters the cytoplasm through transporters. Finally, ASNS converts aspartate to asparagine using glutamine as a nitrogen donor [6]. Glutamine is a non-essential amino acid that plays an important role in cell proliferation and survival, and is involved in the synthesis of other nutrients and various cellular activities [21]. Glutamine deprivation induces cell apoptosis. And it was confirmed that the percentage of living cells was significantly increased when citrate synthase (a TCA cycle enzyme) was knocked down [4]. Citrate synthase (CS) catalyses the formation of citrate from oxaloacetate and acetyl-CoA. This pathway is blocked when CS is inhibited, leading to the transition of oxaloacetate to aspartate and asparagine. This conversion rescues the glutamine-induced apoptosis. Recent studies have shown that glutaminase 1 assembles into a filament-like shape after glutamine deprivation. This shape possesses high activity and substrate-binding affinity, leading to a reduction in intracellular glutamine and, subsequently, intracellular asparagine. Several types of mitogenome-encoded protein (MEPs) synthesis pathways rely on asparagine. Therefore, MEPs will also lack, which further impair electron transfer chain (ETC) function and trigger an outburst of mitochondria-derived reactive oxygen species (ROS) [22]. These signals also result in the intrinsic apoptosis of cells [23]. The addition of asparagine to the medium can restore cell proliferation by preventing ROS burst in long-term glutamine starvation cells, but not alanine, proline, glutamate and aspartate [4, 15]. Glutamine also regulates angiogenesis through multiple mechanisms. The proliferation of endothelial cells (ECs) and vessel sprouting are impaired when exogenous glutamine is not available. At this time, ECs rely on asparagine for proliferation [24]. Asparagine alone can partially rescue ECs defects under low glutamine conditions [25]. Together, these results suggest a critical role for asparagine in cellular adaptation to glutamine deprivation [4]. Asparagine can also exchange extracellular amino acids as an amino acid exchange factor like glutamine. And cells preferentially utilise asparagine as an amino acid exchange factor [26]. Asparagine maintains cell life activities like glutamine, and also seemingly plays a more significant role compared to glutamine because the overconsumption of intracellular asparagine can influence cellular proliferation and induce cell apoptosis, even under glutamine-rich conditions (Fig. 2) [4].

### Adaptive responses to cellular stress

Asparagine is an important regulator of the stress response in cells. The integrated stress response (ISR) of cells is induced by the starvation of various nutrients, such as amino acids and proteins. Upon stimulation, uncharged tRNA binds to general control nonderepressible 2 (GCN2), leading to its dimerization and autophosphorylation. Activated GCN2 phosphorylates eukaryotic translation initiation factor 2 subunit α (eIF2α) to block the initiation of mRNA translation and globally inhibit protein translation. It is also possible to elicit endoplasmic reticulum stress (ERS), also known as the unfolded protein response (UPR). It is initiated by the activation of pancreatic ER kinase (PKR)-like ER kinase (PERK), then phosphorylates eIF2α [27]. As we all know, protein translation is the most energy-consuming process in the cell [28]. The conservation of amino acids and energy would contribute more to cell survival under starvation conditions. However, some specific mRNA targets are efficiently translated after eIF2α phosphorylation. One of these targets is activating transcription factor 4 (ATF4), an important transcriptional factor [29, 30]. ASNS is a target of ATF4. Therefore, ASNS expression increases when cells are subjected to stress, especially asparagine insufficiency. ASNS is used to synthesise asparagine, but not glutamate [26]. The concentrations of the other amino acids did not increase significantly. Asparagine becomes the only urgently needed amino acid in rescue. Moreover, asparagine plays a critical role in restoring protein synthesis, inhibiting ER stress, and reactivating the mammalian target of rapamycin complex (mTORC) signalling pathway in glutamine-deprived ECs [25] (Fig. 2).

### The link between asparagine and cancers

The general metabolism of asparagine has been extensively investigated. However, during the course of the study, investigators found that asparagine metabolism is not only universal, but also specific. Different cancer cells have different signaling pathways that regulate ASNS, and asparagine also play different roles in different cancer types. In the next sections, this review will expound asparagine-specific metabolism in various cancers separately.

### Leukaemia

**Fig. 3:**
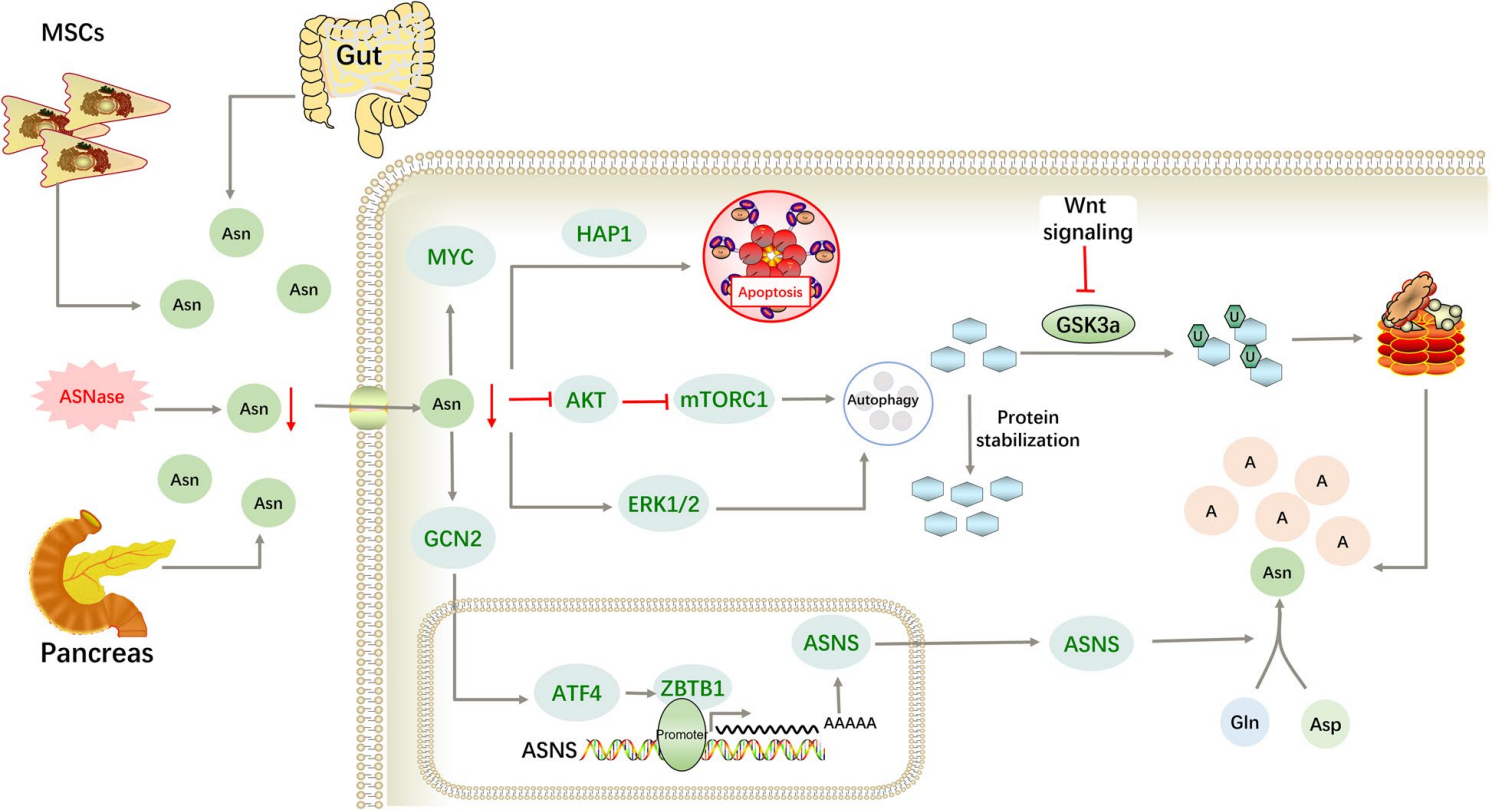
Major mechanisms through which asparagine affects the development of leukaemia. ASNase, a chemotherapeutic drug that targets circulating asparagine, has been approved in the treatment of leukaemia. Asparagine deficiency reduces MYC expression and induces apoptosis in leukemic cells. Leukemic cells initiate a series of stress responses in this situation. Initiation of autophagy, increased expression of ASNS, and decomposition of proteins can increase intracellular asparagine levels. Moreover, asparagine from MSCs, intestinal absorption, and the pancreas increase these levels further. As a result, these compensatory responses decrease the sensitivity of leukemic cells to ASNase

The role of asparagine in leukaemia has been extensively studied (Fig. 3). The prognosis of leukaemia remains poor, with a 5-year survival rate of < 50%. There were 475,000 new cases and 312,000 deaths due to leukaemia worldwide in 2020. Compared to other countries and regions of the world, the overall incidence of leukaemia in China is moderate. In 2020, nearly 62,000 people died of leukaemia in China, corresponding to 19.87% of the global leukaemia deaths. Among those < 18 years, children aged 0–4 years are more likely to develop leukaemia with a higher risk of death [31]. Therefore, it is important to identify effective therapeutic targets.

In 1953, Kidd et al. reported that the serum from guinea pigs could inhibit lymphomas [32]. Then, Broome et al. founded that this inhibition was due to its L-ASNase enzyme activity [33]. ASNS silencing due to ASNS promoter hypermethylation and a high rate of Asn-protein synthesis are observed in most lymphoma cells [34]. For these reasons, ASNase is better at treating leukaemia, especially lymphoblastoma, than other solid cancers. Depletion of asparagine induces growth inhibition and apoptosis in leukaemia cells [35], which is partly dependent on huntingtin-associated protein-1 (HAP1) [36]. Moreover, asparagine depletion reduces MYC protein expression, highlighting the therapeutic potential of suppressing asparagine bioavailability in MYC-driven cancers [37].

Mutations may occur with wider use of ASNase. Leukaemia cells with ASNS gene silencing may begin to express ASNS and acquire resistance to ASNase [38], which depends on the initiation of the nutrient stress response. This is similar to other proliferating cells. After treatment with ASNase, the expression of ATF4 is upregulated by GCN2 kinase [39]. ATF4 then activates Zinc Finger and BTB domain-containing protein 1 (ZBTB1) that is required for leukaemic cell growth under asparagine deprivation. ZBTB1 localises to the nucleus and upregulates ASNS expression by binding to ASNS promoter [40]. ASNS transcript expression is blocked when the ASNS promoter is hypermethylated. Leukaemic cells with high ASNS methylation are more sensitive to ASNase than are cells with low ASNS methylation [41]. Instead, it directly targets CCAAT-enhancer-binding protein homologous protein (CHOP) and subsequently induces programmed cell death independent of ATF4 [42]. Moreover, other adaptation processes are present in ASNase-resistant cells that provide sufficient aspartate and glutamine for the de novo synthesis of asparagine [43]. ASNase was later reported to induce autophagy in leukaemia cells through the AKT/mTORC and extracellular signal-regulated kinase (ERK) signalling pathways [35]. Autophagy enables cells to adapt to nutrient deficiency by regulating the turnover of protein and organelles [44]. Thus, treatments combining ASNase and the suppression of ASNS expression or autophagy have also been explored.

Due to the stress responses in cancer cells, it has been suggested in several studies that ASNS may predict the sensitivity of cancer cells to ASNase. They illustrated that ASNS protein levels play a role in predicting sensitivity, but not ASNS mRNA [45]. However, recent studies have questioned this hypothesis. Lymphoma cells are not dependent on the expression of ASNS as long as there are physiological or supraphysiological concentrations of asparagine in the extracellular milieu [6]. Although ALL cells express low levels of ASNS, mesenchymal stem cells (MSCs) in the cancer microenvironment express high levels of ASNS. MSCs, which exhibited 20-fold higher ASNS levels compared to ALL cells, can protect leukaemic cells from ASNase anti-cancer activity by producing asparagine into the cancer microenvironment [46, 47]. The expression of ASNS in the pancreas also increases after exposure to ASNase, and secreting asparagine into the blood for use by leukaemic cells [27]. Moreover, it was recently reported that gut microbiota can produce amino acids, including asparagine. Intravenously administered ASNase cannot reach the gastrointestinal tract; therefore, it does not influence asparagine in the gut. When the blood asparagine concentration declines, asparagine from the intestinal lumen enters the blood through passive diffusion and interferes with blood asparagine depletion [48]. All of these factors may result in ASNase resistance; therefore, it is important to comprehensively evaluate these indicators when predicting cellular sensitivity to ASNase.

Clinical studies typically utilise the synthetic lethal effects of two substances to identify combination therapy drugs. Synthetic lethality refers to the fact that two non-lethal genes become lethal when simultaneously inactivated. Glycogen synthase kinase 3 (GSK3) associates with ASNase, a synthetic lethal partner in anti-ASNase leukaemic cells. When the Wnt signalling pathway is activated, total cellular protein content and cell size are increased by inhibiting GSK3-dependent protein ubiquitination and proteasomal degradation, which is the Wnt-dependent stabilisation of proteins (Wnt/STOP) [49, 50]. Protein catabolism is a source of asparagine [51]. When ASNase is used to treat leukaemia, proteasomal degradation contributes to maintaining the circulating asparagine levels. Further studies demonstrated that it depends on the N-terminal low-complexity domain of GSK3a. This domain can mediate that GSK3a is segregated to the cytosol together with components of the ubiquitin–proteasome to promote the efficiency of protein degradation. Therefore, inhibition of GSK3a activity can inhibit proteasomal degradation and increase the sensitivity of leukaemic cells to ASNase-focused therapy [52]. Because a compensatory pathway exists in normal cells, the combination of the GSK3 inhibitor and ASNase exhibits few side effects. Moreover, the formation of GSK3a can predict resistance to ASNase in human leukaemia [53].

### Breast cancer

**Fig. 4:**
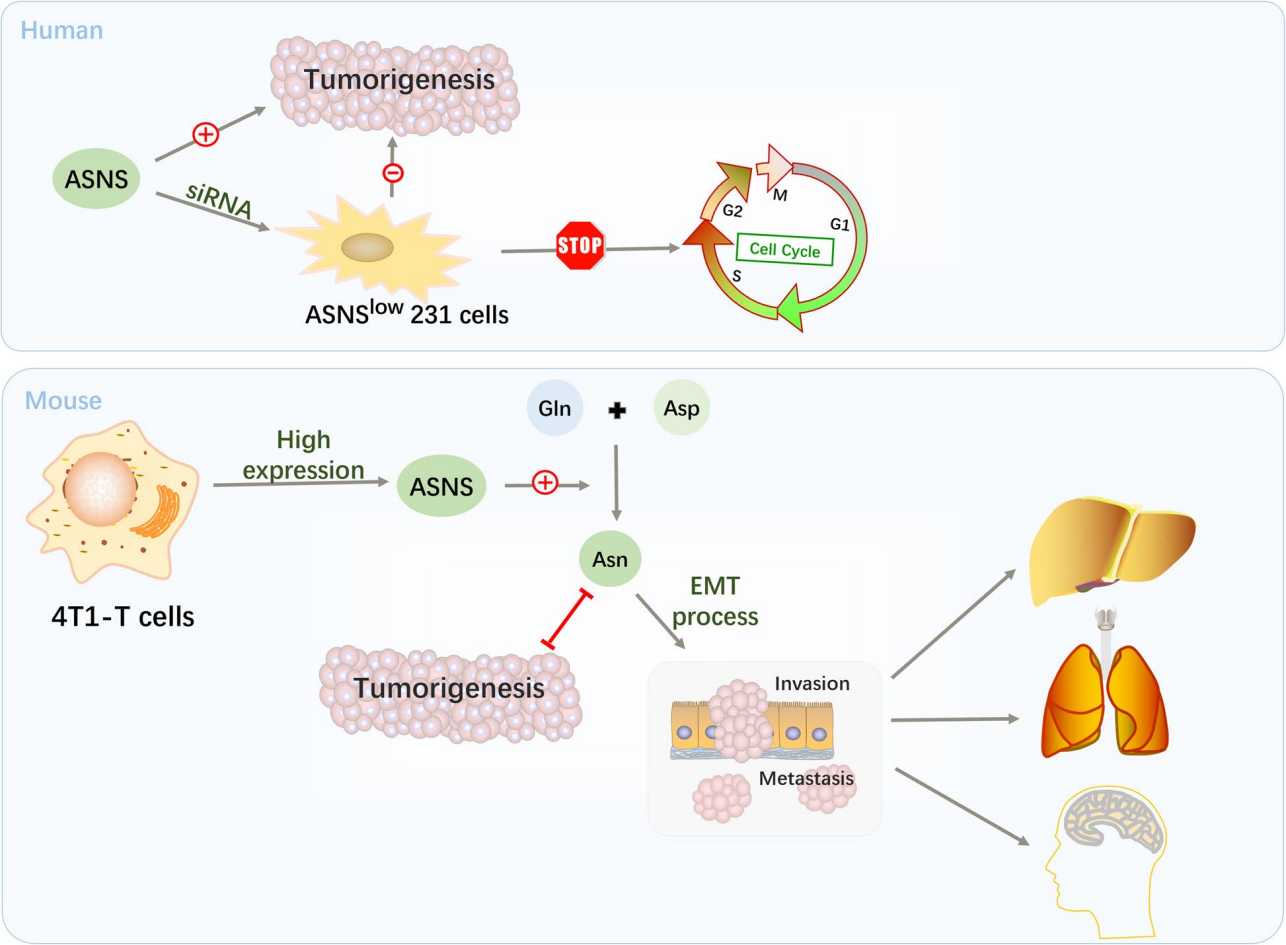
Major mechanisms through which asparagine affects the development of breast cancer. In human breast cancer cells, ASNS expression prompts orthotopic tumour growth. In mouse breast cancer cells, ASNS prompts the EMT process by catalysing the synthesis of asparagine, which results in cancer metastasizing to other organs

The incidence of breast cancer has shown an upward trend and ranks first among female cancers. The global age-standardised incidence of breast cancer is estimated to be 48/100,000 in female. In 2020, there were an estimated 2.26 million cases and nearly 685,000 deaths resulting from breast cancer worldwide, with almost two-thirds of these deaths occurring in less-developed countries. In more developed regions, the overall 5-year survival rate of patients with breast cancer is > 80%, whereas it is < 50% in South Africa. Age is the most important risk factor for breast cancer. In England, more than a third of all instances of breast cancer occur in women aged ≥ 70 years [54]. More than half the patients had locally advanced or metastatic disease at the time of their first diagnosis. Recently, in a study on the relationship between plasma metabolite concentrations and breast cancer risk, the asparagine concentration in the plasma was determined to be negatively associated with cancer risk [55]. Current studies on the function of asparagine in breast cancer have focused on ASNS (Fig. 4). ASNS expression is significantly higher in breast carcinoma tissues than in adjacent normal tissues. Moreover, a MTT assay revealed that ASNS knockdown inhibits the proliferation of breast cancer cells [56]. ASNS expression levels were observed to be positively related to recurrence rate and negatively related to survival [57]. Therefore, they speculated that the ASNS is a valuable prognostic biomarker for breast cancer. Yang et al. generated a human breast cancer cell line with ASNS downregulation. This is the first detailed explanation that the absence of ASNS significantly inhibits the proliferation and colony-forming ability of breast cancer cells, leading to cell cycle arrest in the S phase [58]. Although asparagine is an important precursor for the synthesis of nucleotides [14, 15], further studies are required to determine whether the lack of ASNS inhibits the growth of human-derived breast cancer cells by reducing asparagine levels.

Breast cancer metastasis is a more serious threat than the primary lesions. Patients with breast cancer tend to have metastatic foci in tissues such as the lungs, liver, and brain after the primary cancer is resected. An important characteristic of cancer cells is their high invasive ability. Epithelial-mesenchymal transition (EMT) must be completed when cancer cells metastasise. EMT is a crucial biological process for the metastasis of cancer cells derived from epithelial cells to obtain the ability of metastasis [57]. Some researchers have conducted studies using two mouse breast cancer cell lines (4T1-E and 4T1-T) [59]. These two sublines have different invasiveness, enabling study of the drivers of metastasis. Eleven candidate metastatic driver genes have been identified. Clinical evidence of the correlation between ASNS and cancer development is the most convincing. ASNS expression can predict lung cancer recurrence in patients with breast cancer. Similarly, it was significantly higher in 4T1-T cells, which have a greater potential for metastasis. Compared to a previous study, this study clarified that the effect of ASNS on breast cancer metastasis is attributed to the synthesis of asparagine. Addition of asparagine restored the invasive ability of 4T1-T cells upon ASNS silencing. When circulating asparagine was consumed using ASNase, the metastasis of 4T1-T cells was also evidently decreased compared to the control group. Most 4T1-T cells are in the morphology of epithelial cells during the silencing of ASNS. Asparagine is also a basic component of proteins involved in the EMT process, and asparagine levels selectively increase in proteins that drive EMT. Therefore, we conclude that the effects of asparagine bioavailability on breast cancer metastasis occur at least partly through the regulating of EMT [60]. However, this phenomenon appears to be limited to breast cancer mouse models. This differs from the previously mentioned effect of ASNS on the growth of in situ human breast carcinomas.

Taxanes are some of the most commonly used anticancer drugs in clinical settings. Mechanistically, taxanes directly induce T cells to release cytotoxic extracellular vesicles, which specifically induce apoptosis of cancer cells without affecting healthy cells [61]. Its resistance is typically mediated by the protection of nuclear factor erythroid-2 related factor 2 (Nrf2) against oxidative stress. In a recent clonal transcriptomic analysis of triple-negative breast cancer mice, a lineage with high Nrf2 or taxanes resistance was shown to exert collateral sensitivity against asparagine deprivation through ASNase [62]. These findings provide a theoretical basis for the treatment of breast cancer using ASNase. However, this remains to be further confirmed by clinical studies.

### Melanoma

**Fig. 5:**
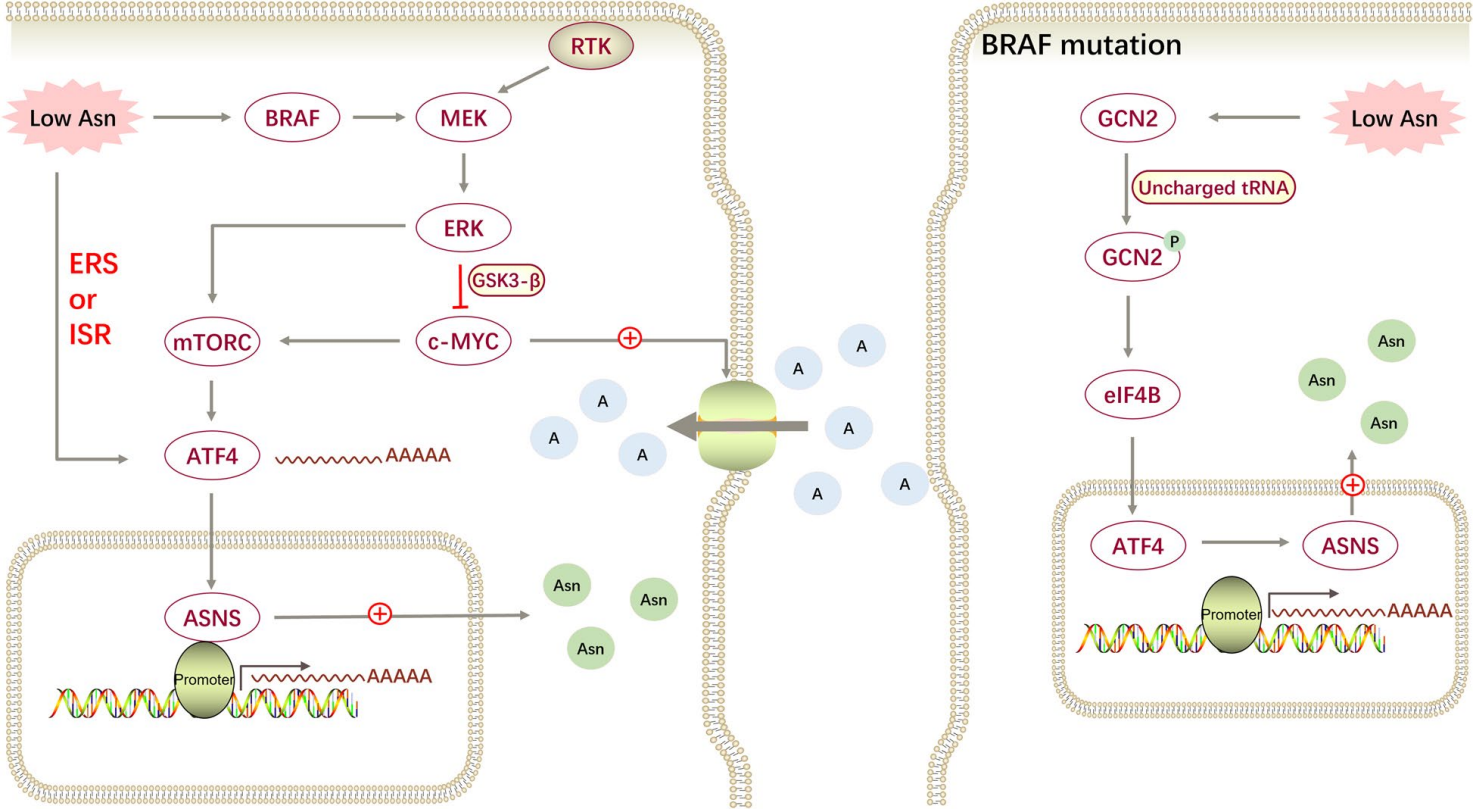
Major mechanisms through which asparagine affects the development of melanoma. Melanoma cells with or without BRAF mutation facilitate the synthesis of asparagine through different stress responses during low asparagine

Melanoma is one of the deadliest forms of skin cancer arising from melanin cells and accounts for 90% of all skin cancer-induced deaths. The incidence of cutaneous melanoma is > 95% of primary melanoma cases. Although its major risk factors are known, the incidence of melanoma doubles every 10 years. According to statistics, the median age at diagnosis is 65 years, and the percentage of female is slightly higher among melanoma patients > 60 years of age [63]. Melanoma cells, compared with normal melanocytes, are highly dependent on glutamine to support their growth. Similarly, asparagine is important for the survival and development of melanoma cells (Fig. 5). The asparagine concentration is elevated in patients with melanoma [64]. The lack of asparagine increases the demand for glutamine, which is an important basis for glutamine addiction in melanoma cells [65]. The expression of ASNS is low in melanoma cells, but is upregulated through a series of auto-compensatory responses during asparagine limitation. In addition to the common ISR and ERS response pathway [66], other compensatory responses have been identified in melanoma. Pathria et al. first determined the role of the mitogen-activated protein kinases (MAPK) pathway activation in limiting asparagine levels [67]. When ASNS is knocked down, the v-raf murine sarcoma viral oncogene homolog B1 (BRAF)-mitogen-activated extracellular signal-regulated kinase (MEK)-ERK-mTORC signalling axis is activated, positively regulating the expression of ATF4 mRNA and its downstream target-ASNS. The receptor tyrosine kinase (RTK) is an upstream regulator of the MAPK pathway signalling, and its activity influences the activation of this pathway activation [68]. Exploiting the synthetic lethality between ASNS and MAPK signalling showed that asparagine depletion combined with the suppression of the MAPK signalling pathway effectively inhibited the growth and metastasis of melanoma in vivo. In a later study, the activated MAPK pathway was discovered to inhibit GSK3-β-mediated c-MYC degradation and consequently, upregulated ATF4. Moreover, elevated c-MYC levels can support melanoma cells in adjusting to asparagine restriction by promoting the uptake of essential amino acids and mTORC activity [68].

A novel stress response was observed in the BRAF-mutated melanoma cells. BRAF mutations are the most common type of mutation in malignant melanoma [69]. In BRAF-mutant melanoma cells, eukaryotic translation initiation factor 4B (eIF4B) induces the transcription and translation of ATF4, which is downstream of the GCN2 regulator, further upregulating ASNS [70]. This novel regulatory pathway mediates the adaptation of cells to stressful conditions. Because the basal expression of ASNS is low in melanoma cells, these cells rely heavily on translational reprogramming pathways that regulate ASNS expression to maintain the asparagine necessary for cell survival. In summary, a combination of inhibiting exogenous asparagine and targeting these pathways is a promising strategy.

### Lung cancer

**Fig. 6:**
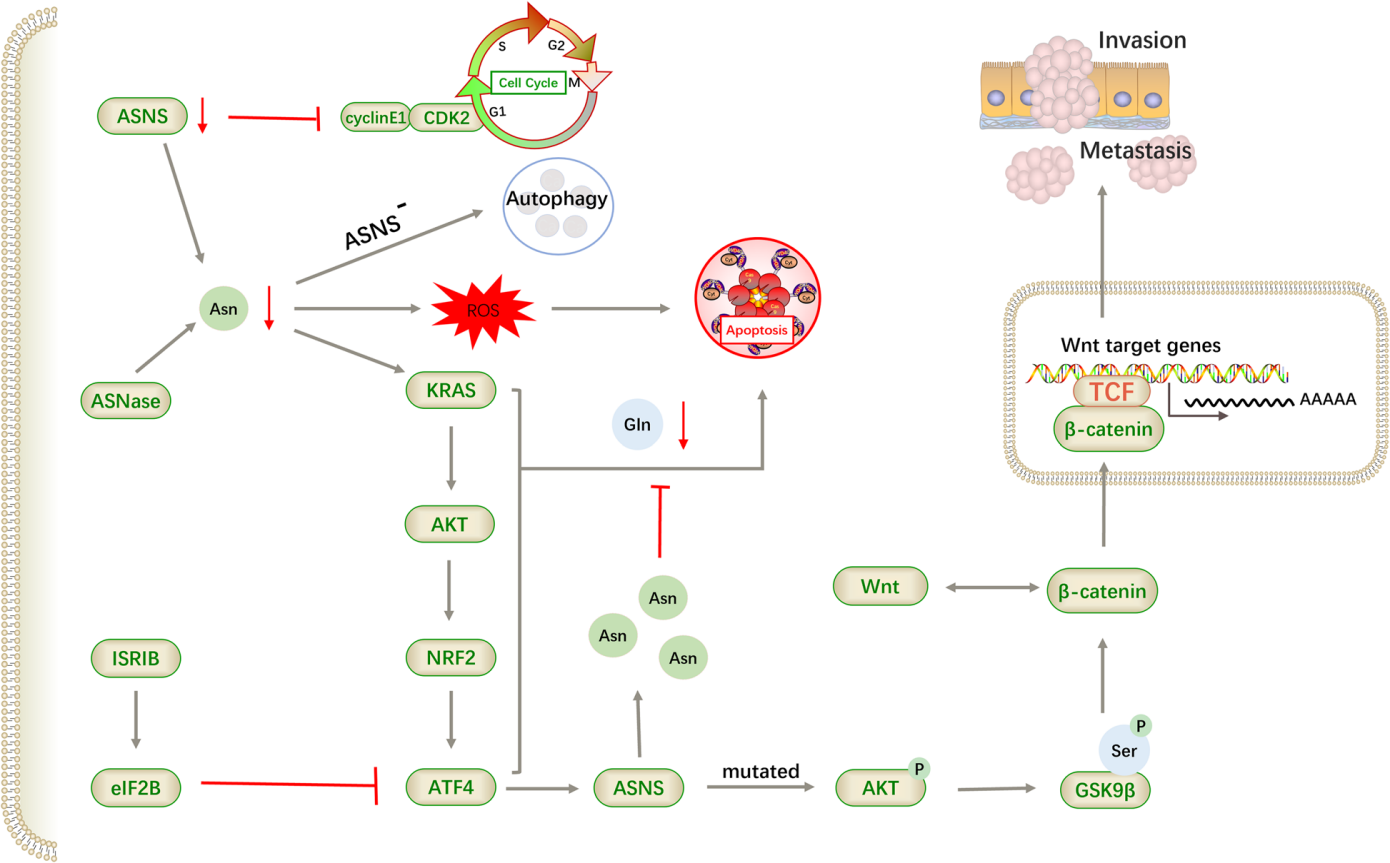
Major mechanisms through which asparagine affects the development of lung cancer. ASNS expression is increased in lung cancer cells and its downregulation directly caused cell-cycle arrest at the G1 phase. The synthesis of asparagine is then reduced, inducing autophagy and apoptosis in lung cancer cells. Under the regulation of KRAS, lung cancer cells promote the synthesis of asparagine by initiating stress responses, which further rescue cells from apoptosis. In addition, lung cancer cells with ASNS mutation cannot synthesize asparagine successfully, but facilitates the invasion and metastasis of lung cancer cells through the AKT-dependent pathway

Lung cancer is the leading cause of death among cancers and accounts for > 20% of all cancer-related deaths. Based on the latest global statistics report (2018), there were 2,093,900 new cases and 1,761,000 new deaths worldwide in 2018. The prognosis of lung cancer remains poor, and the overall 3-year survival rate of patients with non-small-cell lung cancer (NSCLC) after treatment is 31%. Since 2000, the rate of new cases has decreased by an average of 2% annually, and the death rate from lung cancer has declined more dramatically, at an average of 4% annually. In the current study, the estimated mortality rate from lung cancer was 36.7 per 100 000. However, this burden remains significant in the United States and worldwide. The search for the best therapeutic targets for enhancing therapeutic efficacy is ongoing. In recent years, asparagine has drawn increasing attention from researchers (Fig. 6). Asparagine, in combination with several other amino acids, has been proposed to detect early-stage NSCLC [71]. The expression of ASNS was found increased in lung cancer tissues. ASNS knockdown induced cell cycle arrest at the G0/G1 phase by inhibiting the formation of the cyclins CyclinE1-cyclin-dependent kinase 2 (CDK2) complex, which is an important molecule that affects the transition from the G1 to S phase of the cell cycle [72].

NSCLC is the most common type of lung cancer. Asparagine deprivation causes cytotoxicity and apoptosis in NSCLC cells, mediated by ROS. Simultaneously, Kirsten rat sarcoma viral oncogene (KRAS) regulate the nutrient stress response that is initiated during asparagine deprivation in NSCLC. ATF4 is further activated by KRAS and its downstream AKT-NRF2, which promotes asparagine synthesis by upregulating ASNS expression, thus protecting cancer cells from damage and recovering cell clonogenicity and motility. These results indicated the importance of KRAS in maintaining cellular asparagine levels [73]. Other studies were conducted to test the influence of integrated stress response inhibitors (ISRIB) on lung carcinoma cell growth. ISRIB maintains global translation by stabilising eIF2B in the presence of nutrient stress and consequently blocks ATF4 gene translation [74]. However, ISRIB exhibits distinct inhibition of cancer cells only when asparagine is depleted, instead of when serine, glutamine, or glucose are depleted [75]. In contrast, the intensity of nutrient stress and the KRAS- Nrf2-ATF4 signalling axis pathway can trigger apoptosis under conditions such as low glutamine levels. However, the overexpression of asparagine and ASNS inhibits ATF4-dependent apoptosis [73]. Therefore, both ATF4-mediated compensation and apoptosis are dependent on asparagine rescue. To cope with the deletion of asparagine, lung adenocarcinoma cells without ASNS expression initiate autophagic flux, which also occurs in leukaemia cells. Inhibition of autophagy can further increase caspase3-dependent apoptosis [76].

ASNS expression is also strongly associated with cell migration and invasion in lung cancer tissues. It has been described previously that ASNS promoted the metastasis of cancer cells by catalysing the biosynthesis of products-asparagine [57]. However, in lung cancer, relevant studies have shown that high ASNS levels can influence cell invasiveness through an alternative pathway other than asparagine [77]. In terms of mechanism, ASNS activates phosphorylation at Ser3 of GSK9β by phosphorylating Akt to stabilise the β-catenin complex. This signalling axis can regulate the Wnt pathway, which in turn facilitates more β-catenin to translocate into the nucleus. Simultaneously, ASNS increases mitochondrial potential and membrane fusion to regulate mitochondria in response to Wnt stimulation. Therefore, we conclude that even if endogenous asparagine is not produced, ASNS can promote lung cancer cell invasion through the Wnt signalling pathway and mitochondria. After ASNS knockdown, the growth of primary cancers can be maintained by circulating asparagine, whereas cell invasiveness suffers from double impairment: limited asparagine and asparagine-independent pathway disorder.

### Colorectal cancer

Colorectal cancer (CRC), the third leading cause of cancer-related deaths worldwide, accounting for approximately 10% of all cancer-related deaths. Over the past three decades, the number of CRC cases has more than doubled, from 842 098 to 2·17 million, and deaths have increased from 518126 to 109 million [78]. The incidence and mortality of CRC have declined in adults > 50 years of age, whereas the incidence increased from 8.6 per 100,000 in 1992 to 12.9 per 100,000 in 2018 in adults < 50 years of age [79]. Approximately 70–75% of cases occur sporadically, whereas the remaining 25–30% occur in patients with hereditary cancers. Analysis of epidemiological data showed that the 5-year relative survival of patients with CRC increased from 61.5% in the early 1990s to 67.7% in 2010.

**Fig. 7:**
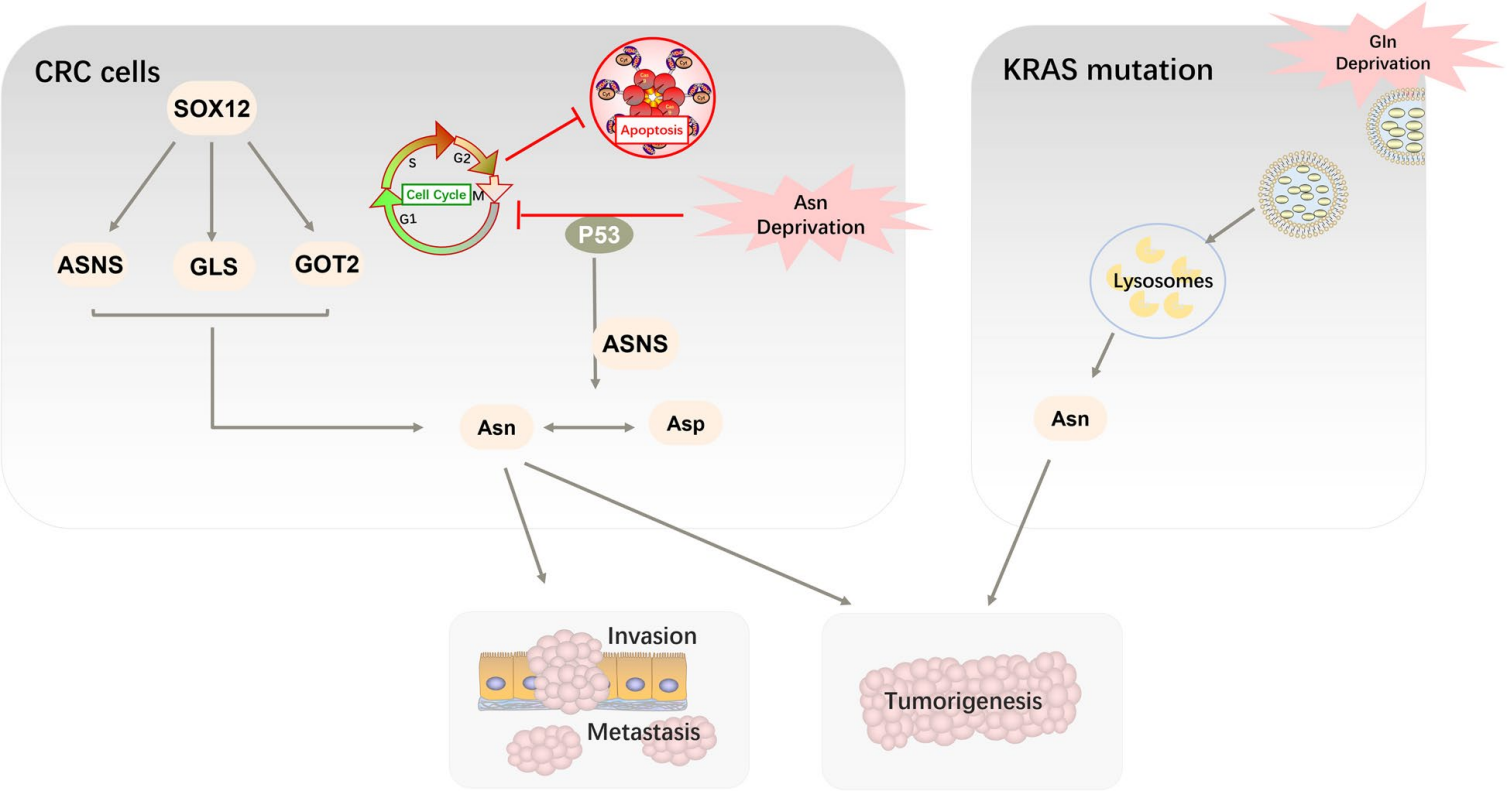
SOX12 and P53 affect the growth and migration of CRC cells by regulating asparagine synthesis. P53 protects CRC cells from apoptosis during asparagine deficiency; therefore, CRC cells with p53 mutations are relatively sensitive to ASNase. In addition, asparagine deficiency induces macropinocytosis in CRC cells with KRAS mutation, providing sufficient asparagine for cells to maintain the proliferation of CRC cells

Metastasis and recurrence of CRC are the leading causes of CRC-related mortality. Based on relevant studies, regular screening can reduce mortality by nearly 50% [80]. Recently, researchers have attempted to explore the mechanism of asparagine in CRC (Fig. 7). In a cohort study correlating the prognosis of CRC, asparagine metabolism was associated with poor survival rates in patients with CRC [81]. In 2019, Du et al. reported that asparagine correlated with poor prognosis‐related genes-sex-determining region Y-box 12 (SOX12), which promoted CRC cell growth and migration in vitro as well as in vivo. Since amino acids are important intermediate metabolites, investigators were prompted to determine whether SOX12 supports CRC cell survival and development by affecting amino acid metabolism. Analysis of amino acids using the RT2 Profiler PCR array showed that ASNS, GLS, and glutamic oxaloacetic transaminase were upregulated upon SOX12 overexpression. All these enzymes are key to produce asparagine, and they have been shown to be involved in SOX12-mediated growth and metastasis of CRC cells. In addition, ASNase inhibits CRC cell proliferation. These results indicate that SOX12 affects cell survival and metastasis in CRC by facilitating asparagine synthesis, specifically under hypoxic conditions [82].

Similar to lung cancer cells, KRAS mutations are also present in CRC cells. Approximately 50% of patients with CRC harbour KRAS mutations [83]. Heterogeneity is a characteristic of malignant tumours and is a barrier to effective cancer treatments. This refers to the generation of daughter cells with distinct molecular biological and genetic characteristics during division. Similar to normal stem cells, CRC cells can divide asymmetrically to generate daughter cell like itself and other daughter cells or progenitor cells, leading to various cell subtypes [84]. Different patients display varying levels of susceptibility to chemotherapeutic drugs because of tumour heterogeneity, and the irrational use of drugs may cause tumour deterioration and metastasis. Therefore, it is helpful to identify the heterogeneity of CRC when searching for suitable targets. CRC can be classified into four subtypes: CMS1, CMS2, CMS3, and CMS4 [85], of which CMS3 is associated with KRAS mutations. It has been shown that CRC cells with KRAS mutations adapt to glutamine depletion through the biosynthesis of asparagine [86]. Subsequently, asparagine was linked to macropinocytosis following nutrient deficiency. Nutrient deficiency can induce macropinocytosis and melanocytosis in CRC cells with KRAS mutations. Macropinocytosis, an actin-dependent endocytic process, internalises extracellular proteins, such as albumin, that are further degraded within lysosomes, which provide a variety of amino acids to meet the nutrient demands of developing cancer [87]. Of these, asparagine was also included. Upon glutamine depletion, ASNS knockdown accelerates macropinocytosis. Inhibition of macropinocytosis and the ASNS gene alone reduced the growth rate of CRC cells, but the combination of both almost completely suppressed cancer growth. The combination group showed no observed effect on mouse weight [88]. This may be a promising novel strategy for the treatment of KRAS mutations.

In addition to KRAS mutations, p53 mutations are another common type of mutation in CRC, and patients with p53 mutations experience poor treatment outcomes. P53 participates widely in various anti-proliferative reactions as a cancer suppressor. A recent study revealed a novel association between asparagine and p53. Under physiological conditions, P53 can bind to ASNS and inhibit its expression, which regulates homeostasis between aspartic acid and asparagine. In turn, decreased asparagine is perceived by LKB1, which then activates MAPK and subsequently induces p53-dependent cell cycle arrest, thereby protecting the cells from apoptosis. Moreover, apoptosis of p53-null CRC cells increased in the absence of asparagine due to increased asparagine sensitivity [89]. Therefore, in CRC cells with p53 mutations, asparagine limitation provides drug therapy, making CRC cells more sensitive to radiotherapy [90].

These findings suggest that some critical genes that influence the development of CRC correlate with asparagine metabolism, thus providing further possibilities for the use of asparagine deficiency in the treatment of CRC.

### ASNase therapy

ASNase, a chemotherapeutic agent targeting free asparagine, has been approved for cancer treatment and has achieved a certain degree of success in the clinical treatment of patients with leukaemia. The combination treatment with ASNase and vindesine, as well as combined treatment with ASNase and prednisone can induce remission in up to 90% of children with ALL. And ASNase may enhance the sensitivity of cancer cells to radiotherapy. ASNase is generally well tolerated by most patients, and few patients develop an anaphylactic reaction or anti-ASNase antibodies when they initially receive ASNase [91]. High ASNase activity in the blood can effectively prevent central nervous system relapse and improve prognosis. ASNase is an ideal chemotherapeutic drug even for infants with leukaemia. In one study, almost all children with ALL achieved a complete remission after ASNase treatment [92]. The treatment effect of ASNase depends on the asparagine consumption level as well as on the corresponding duration. Any residual asparagine can cause treatment failure or cancer recurrences [93]. Therefore, it is beneficial to combine therapeutic drug monitoring (TDM) with ASNase treatment [94]. In children with B-acute lymphoblastic leukaemia (B-ALL), the levels of asparagine in the plasma and bone marrow showed a strong correlation, whereas there was no significant correlation between the plasma and cerebrospinal fluid (CSF) [95, 96]. Depletion of asparagine in the CSF helps reduce central nervous system involvement; therefore, it can achieve the best monitoring effects to simultaneously measure asparagine in both plasma and CSF. However, the measurement of plasma asparagine concentrations is more frequently performed because it is difficult to obtain CSF. In addition to the direct measurement of asparagine, we can also determine ASNase activity to monitor asparagine depletion in the serum [97].

However, ASNase may introduce undesirable side effects such as thrombus, hypersensitivity, hyperglycemia, hypertriglyceridemia, acute pancreatitis, and hepatotoxicity [20, 92, 98, 99]. For decades, researchers continually improve ASNase structure, in order to increase treatment outcomes and reduce side effects. Native E. coli L-ASNase and pegaspargase are major components of ALL treatment regimens. At present, many modified asparaginases have appeared. For example, the recombinant L-ASNase from the genus Anoxybacillus possesses good thermal stability without glutaminase activity [100]. And L-ASNase GRASPA (®), which is encapsulated in red blood cells, is well tolerated and reduces the occurrence of allergic reactions and coagulation disorders [101]. However, almost all of these ASNase studies are based on leukaemia cells. If we want to use it for the treatment of other solid cancers, further in vitro and in vivo studies may be needed to evaluate its actual effectiveness in other cancers. Moreover, with the extensive study of asparagine, asparagine metabolism in cancer is gradually being unveiled. Thus, combination of ASNase with asparagine metabolism will achieve more precise treatment.

## Discussion

Cancer is the second most common cause of death and a worldwide threat to human health. Clinical researchers are continuously seeking effective methods and medicine of treatments. Cancer cells have a relative nutrient deficiency because of their high metabolic rates. They often sustain their survival and development through various metabolic reprogramming processes [102], which are considered specific hallmarks of cancer [103]. First, these stress processes are considered to produce more glutamine [88]. However, an increasing number of studies have shown that cancer cell growth is dependent on asparagine. In mammals, asparagine is not broken down but is primarily involved in protein translation. Asparagine-mediated protein translation is necessary for the proliferation and migration of adaptive cells [75]. Interestingly, asparagine also regulates senescence. For example, during glutamine deficiency, p53-dependent senescence was reversed by asparagine supplementation [89].

Metabolomic analysis is a useful method for identifying clinically meaningful biomarkers and treatment targets and is widely used in the field of cancer research [104]. Metabolomic analysis have shown that asparagine is closely related to cancer progression and metastasis. Therefore, asparagine targeting has gradually become a promising strategy for cancer treatment. Depletion of circulating asparagine stimulates cells to initiate stress signalling cascades and upregulates ASNS gene expression. Although these stress responses eventually promote ASNS expression, there are different regulatory programs in response to nutritional stress response in cancer cells. By fully understanding the metabolic processes of asparagine in different cancers, we can select specific inhibitors to block these compensatory pathways. Then, the source of asparagine is further cut off, which is more effective in inhibiting cancer cells and improve clinical outcomes especially for solid cancers that are not sensitive to ASNase alone. There is much experimental evidence indicated these combined treatments particularly effective.

In the previous content, we have reviewed not only asparagine metabolism in several cancers but also in the case of certain gene mutations, which provide many targets for cancer therapy (Table 1). However, there are still some limitations. Including leukaemia, there was no clear evidence demonstrates ASNS expression levels predict the sensitivity of cancer cells to ASNase. Moreover, in human-derived breast cancer cell lines, we cannot conclude that ASNS promotes cancer progression by upregulating asparagine levels. And ASNS can influence lung cell invasiveness through an alternative pathway other than asparagine. There have been inhibitors that directly target ASNS [23]. Perhaps this allows us to confuse the relationship between ASNS and asparagine. But this also further illustrates the metabolism specificity of asparagine in cancer cells and adds depth to the discussion.

**Table 1:** Signal molecules and processes related to asparagine metabolism in various cancers

**Cancer type**	**Signal molecules and processes related to asparagine metabolism (potential therapeutic targets)**	**References**
**Leukaemia**	HAP1	[36]
	MYC	[37]
	GCN2- ATF4	[39]
	ATF4-ZBTB1	[40]
	CHOP	[42]
	AKT/mTORC- autophagy	[35]
	ERK- autophagy	[35]
	GSK3	[52]
**Breast cancer**	cell cycle	[58]
	EMT	[60]
	Nrf2	[62]
**Melanoma**	BRAF-MEK-ERK-mTORC-ATF4	[67]
	RTK	[68]
	c-MYC- mTORC	[68]
	GCN2-eIF4B-ATF4	[70]
**Lung cancer**	cyclins CyclinE1-CDK2 complex	[72]
	ROS- apoptosis	[73]
	KRAS- AKT-NRF2-ATF4	[73]
	eIF2B	[74]
	autophagy	[76]
**CRC**	SOX12	[82]
	macropinocytosis	[88]
	p53	[89]

Immunotherapy has become one of the most important therapeutic strategies for treating cancers, which has greatly promoted the progress of cancer treatment. The immune checkpoint blockade (ICB) based on monoclonal antibodies targeting immune checkpoint proteins and adoptive cell transfer (ACT) based on tumour-infiltrating lymphocytes or CAR T cells are frontline cancer immunotherapies. Recently, researchers have begun to relate asparagine and immunity. In the early stages of antigen stimulation, asparagine induces the transition of naïve CD8 + T lymphocytes to an active state by phosphorylating lymphocyte-specific protein tyrosine kinase (Lck) [105]. While for activated CD8 + T lymphocytes, asparagine restriction can enhance CD8 + T cell metabolic fitness and antitumoral functionality through the Nrf2-dependent stress response. In preclinical animal models, the combination of asparagine restriction with anti-PD-L1 antibodies displayed a better anti-tumour effect than the anti-PD-L1 monotherapy alone group [106]. These results suggested that Asn restriction is a promising and clinically relevant strategy to enhance cancer immunotherapy against multiple cancer types.

## Conclusion

After decades of research on asparagine, its essential role in mammals cannot be ruled out. Asparagine is essential for cancer growth and development. It can participate in the metabolism of other intracellular nutrients via mTORC1 signaling cascade, maintaining the nutrient demand of cancer cells, and promote cancer metastasis by influencing EMT pathway. Once asparagine is insufficient, cancer cells can activate ISR and ERS to upregulate the expression of ASNS to synthesize sufficient asparagine. Moreover, for different cancer cells, there are also different programs to regulate asparagine metabolism, but ultimately lead to increased expression of ASNS. Based on the importance of asparagine in cancer cells, ASNase targeting asparagine has been used for the treatment of leukaemia. However, the treatment effect of ASNase in other solid cancers is not good, mainly because of the mechanism of reprogramming asparagine metabolism. With the roles of asparagine in the physiological state and stress response are gradually explored, this limitation will hopefully be addressed in the future. By combining inhibition of ASNS or inhibition of targets that regulate ASNS with asparagine restriction, the level of asparagine will be greatly reduced and the growth of cancer cells will be inhibited. At present, there are two methods of asparagine restriction: dietary restriction and ASNase treatment. ASNase is being refined to improve efficacy and reduce side effects. In addition, the combination of asparagine restriction with radiotherapy and immunotherapy has also begun to become a new cancer treatment strategy. Although there is preliminary experimental evidence that revealed the efficacy of the combination therapy, more experimental data are needed to support it. In the future, asparagine remains an ideal target for the strategy of nutrient restriction.

## Data Availability

No datasets were generated or analysed during the current study..

## Abbreviations

4E-BP1: EIF4E-binding protein 1
ACT: Adoptive cell transfer
ARF1: ADP-ribosylation factor 1
ASNase: Asparaginase
ASNS: Asparagine synthase
ATF4: Activating transcription factor 4
B-ALL: B-acute lymphoblastic leukaemia
BAT: Brown adipose tissue
BRAF: V-raf murine sarcoma viral oncogene homolog B1
CAD: Carbamoyl-phosphate synthetase 2, aspartate transcarbamoylase, dihydroorotatase
CDK2: Cyclin-dependent kinase 2
CHOP: CCAAT-enhancer-binding protein homologous protein
CRC: Colorectal cancer
CS: Citrate synthase
CSF: Cerebrospinal fluid
ECs: Endothelial cells
eIF2α: Eukaryotic translation initiation factor 2 subunit α
eIF4B: Eukaryotic translation initiation factor 4B
eIF4E: Eukaryotic translation initiation factor 4E
EMT: Epithelial-mesenchymal transition
ERK: Extracellular signal-regulated kinase
ERS: Endoplasmic reticulum stress
ETC: Electron transfer chain
GCN2: General control nonderepressible 2
Glut1: Glucose transporter type 1
Glut4: Glucose transporter type 4
GSK3: Glycogen synthase kinase 3
HAP1: Huntingtin-associated protein-1
HK2: Hexokinase 2
ICB: Immune checkpoint blockade
ISR: Integrated stress response
ISRIB: Integrated stress response inhibitors
KRAS: Kirsten rat sarcoma viral oncogene
Lck: Lymphocyte-specific protein tyrosine kinase
MAPK: Mitogen-activated protein kinase
MEK: Mitogen-activated extracellular signal-regulated kinase
MEPs: Mitogenome-encoded proteins
MSCs: Mesenchymal stem cells
mTORC: Mammalian target of rapamycin complex
mTORC1: Mammalian target of rapamycin complex 1
MYC: Myelocytomatosis oncogene
Nrf2: Nuclear factor erythroid-2 related factor 2
NSCLC: Non-small-cell lung cancer
PERK: Pancreatic ER kinase (PKR)-like ER kinase
Pkm: Pyruvate kinase
ROS: Reactive oxygen species
RTK: Receptor tyrosine kinase
S6K1: S6 kinase 1
SOX12: Sex-determining region Y-box 12
TCA cycle: Tricarboxylic acid cycle
TDM: Therapeutic drug monitoring
UPR: Unfolded protein response
Wnt/STOP: Wnt-dependent stabilization of proteins
ZBTB1: Zinc Finger and BTB domain-containing protein 1

## References

[1] Yuneva M, Zamboni N, Oefner P, Sachidanandam R, Lazebnik Y (2007). Deficiency in glutamine but not glucose induces MYC-dependent apoptosis in human cells. J Cell Biol.

[2] Pathria G, Lee JS, Hasnis E, Tandoc K, Scott DA, Verma S, Feng Y, Larue L, Sahu AD, Topisirovic I (2019). Translational reprogramming marks adaptation to asparagine restriction in cancer. Nat Cell Biol.

[3] Birsoy KW, Chen T, Freinkman WW, Abu-Remaileh M, Sabatini DM (2015). An essential role of the mitochondrial electron transport chain in cell proliferation is to enable aspartate synthesis. Cell.

[4] Zhang J, Fan J, Venneti S, Cross JR, Takagi T, Bhinder B, Djaballah H, Kanai M, Cheng EH, Judkins AR (2014). Asparagine plays a critical role in regulating cellular adaptation to glutamine depletion. Mol Cell.

[5] Albertsen BK, Grell K, Abrahamsson J, Lund B, Vettenranta K, Jonsson OG, Frandsen TL, Wolthers BO, Heyman M, Schmiegelow K (2019). Intermittent versus continuous PEG-Asparaginase to reduce asparaginase-associated toxicities: a NOPHO ALL2008 randomized study. J Clin Oncol.

[6] Manuel Grima-Reyes AV, Ivan Nemazanyy, Pauline Meola, Rachel Paul, , Julie Reverso-Meinietti AM-T, Nicolas Nottet, Wai-Kin Chan, , Philip L. Lorenzi SM, Jean-Ehrland Ricci, Johanna Chiche: Tumoral microenvironment prevents de novo asparagine biosynthesis in B cell lymphoma, regardless of ASNS expression. Science advances 2022, 8(27):eabn6491. 10.1126/sciadv.abn6491 PMC9258813 35857457

[7] Mayers JR, Vander Heiden MG (2015). Famine versus feast: understanding the metabolism of tumors in vivo. Trends Biochem Sci.

[8] Krall AS, Mullen PJ, Surjono F, Momcilovic M, Schmid EW, Halbrook CJ, Thambundit A, Mittelman SD, Lyssiotis CA, Shackelford DB (2021). Asparagine couples mitochondrial respiration to ATF4 activity and tumor growth. Cell Metab.

[9] Weintraub SJ, Deverman BE (2007). Chronoregulation by asparagine deamidation. Sci STKE.

[10] Wang JRS, Saei AA, Zhang X, Zubarev RA (2022). First experimental evidence for reversibility of ammonia loss from asparagine. Int J Mol Sci.

[11] Bergström T, Fredriksson SÅ, Nilsson C, Åstot C (2015). Deamidation in ricin studied by capillary zone electrophoresis- and liquid chromatography-mass spectrometry. J Chromatogr B Analyt Technol Biomed Life Sci.

[12] Meng D, Yang Q, Wang H, Melick CH, Navlani R, Frank AR, Jewell JL (2020). Glutamine and asparagine activate mTORC1 independently of Rag GTPases. J Biol Chem.

[13] Duvel K, Yecies JL, Menon S, Raman P, Lipovsky AI, Souza AL, Triantafellow E, Ma Q, Gorski R, Cleaver S (2010). Activation of a metabolic gene regulatory network downstream of mTOR complex 1. Mol Cell.

[14] Ben-Sahra I, Howell JJ, Asara JM, Manning BD (2013). Stimulation of de novo pyrimidine synthesis by growth signaling through mTOR and S6K1. Science.

[15] Zhu Y, Li T, Ramos da Silva S, Lee JJ, Lu C, Eoh H, Jung JU, Gao SJ, Meng XJ, Longnecker R (2017). A critical role of glutamine and asparagine γ-nitrogen in nucleotide biosynthesis in cancer cells hijacked by an oncogenic virus. Bio.

[16] Laplante M, Sabatini DM (2012). mTOR signaling in growth control and disease. Cell.

[17] Lidell ME, Betz MJ, Dahlqvist Leinhard O, Heglind M, Elander L, Slawik M, Mussack T, Nilsson D, Romu T, Nuutila P (2013). Evidence for two types of brown adipose tissue in humans. Nat Med.

[18] Nguyen HP, Yi D, Lin F, Viscarra JA, Tabuchi C, Ngo K, Shin G, Lee AYF, Wang Y, Sul HS (2020). Aifm2, a NADH oxidase, supports robust glycolysis and is required for cold- and diet-induced thermogenesis. Molecular Cell.

[19] Winther S, Isidor MS, Basse AL, Skjoldborg N, Cheung A, Quistorff B, Hansen JB (2018). Restricting glycolysis impairs brown adipocyte glucose and oxygen consumption. Am J Physiol Endocrinol Metab.

[20] Xu YS, Cui T, Yan X, Wang L, Xu Q, Zhao X, Xu Q, Tang X, Tang QQ, Pan HD (2021). Asparagine reinforces mTORC1 signaling to boost thermogenesis and glycolysis in adipose tissues. EMBO J.

[21] Nicklin P, Bergman P, Zhang B, Triantafellow E, Wang H, Nyfeler B, Yang H, Hild M, Kung C, Wilson C (2009). Bidirectional transport of amino acids regulates mTOR and autophagy. Cell.

[22] Zoncu R, Efeyan A, Sabatini DM (2011). mTOR: from growth signal integration to cancer, diabetes and ageing. Nat Rev Mol Cell Biol.

[23] Zhu W, Radadiya A, Bisson C, Wenzel S, Nordin BE, Martinez-Marquez F, Imasaki T, Sedelnikova SE, Coricello A, Baumann P (2019). High-resolution crystal structure of human asparagine synthetase enables analysis of inhibitor binding and selectivity. Commun Biol.

[24] Pavlova NN, Hui S, Ghergurovich JM, Fan J, Intlekofer AM, White RM, Rabinowitz JD, Thompson CB, Zhang J (2018). As extracellular glutamine levels decline, asparagine becomes an essential amino acid. Cell Metab.

[25] Huang H, Vandekeere S, Kalucka J, Bierhansl L, Zecchin A, Bruning U, Visnagri A, Yuldasheva N, Goveia J, Cruys B (2017). Role of glutamine and interlinked asparagine metabolism in vessel formation. EMBO J.

[26] Krall AS, Xu S, Graeber TG, Braas D, Christofk HR (2016). Asparagine promotes cancer cell proliferation through use as an amino acid exchange factor. Nat Commun.

[27] Mukherjee AAN, Rose FT, Ahmad AN, Javed TA, Wen L, Bottino R, Xiao X, Kilberg MS, Husain SZ (2020). Asparagine Synthetase Is Highly Expressed at Baseline in the Pancreas Through Heightened PERK Signaling. Cell Mol Gastroenterol Hepatol.

[28] Lahtvee PJSB, Smialowska A, Kasvandik S, Elsemman IE, Gatto F, Nielsen J (2017). Absolute quantification of protein and mRNA abundances demonstrate variability in gene-specific translation efficiency in yeast. Cell Syst.

[29] Balsa ESM, Thomas A, Cogliati S, García-Poyatos C, Martín-García E, Jedrychowski M, Gygi SP, Enriquez JA, Puigserver P (2019). ER and nutrient stress promote assembly of respiratory chain supercomplexes through the PERK-eIF2alpha Axis. Molecular Cell.

[30] B’chir W, Maurin AC, Carraro V, Averous J, Jousse C, Muranishi Y, Parry L, Stepien G, Fafournoux P, Bruhat A (2013). The pathway is essential for stress-induced autophagy gene expression. Nucleic Acids Res.

[31] Lin K, Jia H, Cao M, Xu T, Chen Z, Song X, Miao Y, Yao T, Dong C, Shao J (2023). Epidemiological characteristics of leukemia in China, 2005–2017: a log-linear regression and age-period-cohort analysis. BMC Public Health.

[32] Kidd JG (1953). Regression of transplanted lymphomas induced in vivo by means of normal guinea pig serum. I. Course of transplanted cancers of various kinds in mice and rats given guinea pig serum, horse serum, or rabbit serum. J Exp Med.

[33] Broome JD (1963). Evidence that the L-asparaginase of guinea pig serum is responsible for its antilymphoma effects. I. Properties of the L-asparaginase of guinea pig serum in relation to those of the antilymphoma substance. J Exp Med.

[34] Touzart A, Lengliné E, Latiri M, Belhocine M, Smith C, Thomas X, Spicuglia S, Puthier D, Pflumio F, Leguay T, Graux C, Chalandon Y, Huguet F, Leprêtre S, Ifrah N, Dombret H, Macintyre E, Hunault M, Boissel N, Asnafi VA (2019). epigenetic silencing affects l-asparaginase sensitivity and predicts outcome in T-ALL. Clin Cancer Res.

[35] Song PYL, Fan J, Li Y, Zeng X, Wang Z, Wang S, Zhang G, Yang P, Cao Z, Ju D (2015). Asparaginase induces apoptosis and cytoprotective autophagy in chronic myeloid leukemia cells. Oncotarget.

[36] Lee JK, Kang S, Wang X, Rosales JL, Gao X, Byun HG, Jin Y, Fu S, Wang J, Lee KY (2019). HAP1 loss confers l-asparaginase resistance in ALL by downregulating the calpain-1-Bid-caspase-3/12 pathway. Blood.

[37] Srivastava S, Jiang J, Misra J, Seim G, Staschke KA, Zhong M, Zhou L, Liu Y, Chen C, Dave U (2022). Asparagine bioavailability regulates the translation of MYC oncogene. Oncogene.

[38] Ding Y, Li Z, Broome JD (2005). Epigenetic changes in the repression and induction of asparagine synthetase in human leukemic cell lines. Leukemia.

[39] Darvishi F, Faraji N, Shamsi F (2019). Production and structural modeling of a novel asparaginase in Yarrowia lipolytica. Int J Biol Macromol.

[40] Williams RTGR, Gates LA, Barrows D, Passarelli MC, Carey B, Baudrier L, Jeewajee S, La K, Prizer B, Malik S, Garcia-Bermudez J, Zhu XG, Cantor J, Molina H, Carroll T, Roeder RG, Abdel-Wahab O, Allis CD, Birsoy K (2020). ZBTB1 Regulates Asparagine Synthesis and Leukemia Cell Response to L-Asparaginase. Cell Metab.

[41] Akahane K, Kimura S, Miyake KW, Atsushi Kagami, Keiko Yoshimura, Kentaro, Shinohara T, Harama D, Kasai SG, Kumiko Kawai, Tomoko Hata, Kenichiro Kiyokawa, Nobutaka Koh, Katsuyoshi Imamura, Toshihiko Horibe, Keizo Look, A. Thomas Minegishi, Masayoshi Sugita, Kanji Takita, Junko, Inukai T: Association of allele-specific methylation of the ASNS gene with asparaginase sensitivity and prognosis in T-ALL. Blood Advances 2022, 6(1):212–224. 10.1182/bloodadvances.2021004271 PMC8753197 34535013

[42] Jiang J, Srivastava S, Seim G, Pavlova NN, King B, Zou L, Zhang C, Zhong M, Feng H, Kapur R (2019). Promoter demethylation of the asparagine synthetase gene is required for ATF4-dependent adaptation to asparagine depletion. J Biol Chem.

[43] Ehsanipour EA, Sheng X, Behan JW, Wang X, Butturini A, Avramis VI, Mittelman SD (2013). Adipocytes cause leukemia cell resistance to L-asparaginase via release of glutamine. Cancer Res.

[44] Pampliega O, Orhon I, Patel B, Sridhar S, Diaz-Carretero A, Beau I, Codogno P, Satir BH, Satir P, Cuervo AM (2013). Functional interaction between autophagy and ciliogenesis. Nature.

[45] Su N, Pan YX, Zhou M, Harvey RC, Hunger SP, Kilberg MS (2008). Correlation between asparaginase sensitivity and asparagine synthetase protein content, but not mRNA, in acute lymphoblastic leukemia cell lines. Pediatr Blood Cancer.

[46] Iwamoto S, Mihara K, Downing JR, Pui CH, Campana D (2007). Mesenchymal cells regulate the response of acute lymphoblastic leukemia cells to asparaginase. J Clin Invest.

[47] Steiner M, Hochreiter D, Kasper DC, Kornmuller R, Pichler H, Haas OA, Potschger U, Hutter C, Dworzak MN, Mann G (2012). Asparagine and aspartic acid concentrations in bone marrow versus peripheral blood during Berlin-Frankfurt-Munster-based induction therapy for childhood acute lymphoblastic leukemia. Leuk Lymphoma.

[48] Dunn KA, Forbrigger Z, Connors J, Rahman M, Cohen A, Van Limbergen J, Langille MGI, Stadnyk AW, Bielawski JP, Penny SL (2021). Gut bacterial gene changes following pegaspargase treatment in pediatric patients with acute lymphoblastic leukemia. Leuk Lymphoma.

[49] Taelman VF, Dobrowolski R, Plouhinec JL, Fuentealba LC, Vorwald PP, Gumper I, Sabatini DD, De Robertis EM (2010). Wnt signaling requires sequestration of glycogen synthase kinase 3 inside multivesicular endosomes. Cell.

[50] Hinze LLR, Degar J, Han T, Schatoff EM, Schreek S, Karim S, McGuckin C, Sacher JR, Wagner F, Stanulla M, Yuan C, Sicinska E, Giannakis M, Ng K, Dow LE, Gutierrez A (2020). Exploiting the therapeutic interaction of WNT pathway activation and asparaginase for colorectal cancer therapy. Cancer Discov.

[51] Suraweera A, Munch C, Hanssum A, Bertolotti A (2012). Failure of amino acid homeostasis causes cell death following proteasome inhibition. Mol Cell.

[52] Hinze LPM, Karim S, Degar J, McGuckin C, Vinjamur D, Sacher J, Stevenson KE, Neuberg DS, Orellana E, Stanulla M, Gregory RI, Bauer DE, Wagner FF, Stegmaier K, Gutierrez A (2019). Synthetic lethality of wnt pathway activation and asparaginase in drug-resistant acute leukemias. Cancer Cell.

[53] Panetta JC, Liu Y, Bottiglieri T, Arning E, Cheng C, Karol SE, Yang JJ, Zhou Y, Inaba H, Pui CH (2021). Pharmacodynamics of cerebrospinal fluid asparagine after asparaginase. Cancer Chemother Pharmacol.

[54] Wilkinson L, Gathani T (2022). Understanding breast cancer as a global health concern. Br J Radiol.

[55] His M, Viallon V, Dossus L, Gicquiau A, Achaintre D, Scalbert A, Ferrari P, Romieu I, Onland-Moret NC, Weiderpass E (2019). Prospective analysis of circulating metabolites and breast cancer in EPIC. BMC Med.

[56] Qin C, Yang X, Zhan Z (2020). High expression of asparagine synthetase is associated with poor prognosis of breast cancer in Chinese population. Cancer Biother Radiopharm.

[57] Luo M, Brooks M, Wicha MS (2018). Asparagine and glutamine: co-conspirators fueling metastasis. Cell Metab.

[58] Yang H, He X, Zheng Y, Feng W, Xia X, Yu X, Lin Z (2014). Down-regulation of asparagine synthetase induces cell cycle arrest and inhibits cell proliferation of breast cancer. Chem Biol Drug Des.

[59] Wagenblast E, Soto M, Gutierrez-Angel S, Hartl CA, Gable AL, Maceli AR, Erard N, Williams AM, Kim SY, Dickopf S (2015). A model of breast cancer heterogeneity reveals vascular mimicry as a driver of metastasis. Nature.

[60] Knott SRV, Wagenblast E, Khan S, Kim SY, Soto M, Wagner M, Turgeon MO, Fish L, Erard N, Gable AL (2018). Asparagine bioavailability governs metastasis in a model of breast cancer. Nature.

[61] Vennin C, Cattaneo CM, Bosch L, Vegna S, Ma X, Damstra HGJ, Martinovic M, Tsouri E, Ilic M, Azarang L (2023). Taxanes trigger cancer cell killing in vivo by inducing non-canonical T cell cytotoxicity. Cancer Cell.

[62] Nicholls A, Kania K, Bressan D, Hannon GJ, Sawicka K, Wild SA CI, CRUK IMAXT Grand Challenge Team (2022). Clonal transcriptomics identifies mechanisms of chemoresistance and empowers rational design of combination therapies. elife.

[63] Purim KSM, Bonetti JPC, Silva JYF, Marques LB, Pinto MCS, Ribeiro LC (2020). Characteristics of melanoma in the elderly. Rev Col Bras Cir.

[64] Muqaku B, Eisinger M, Meier SM, Tahir A, Pukrop T, Haferkamp S, Slany A, Reichle A, Gerner C (2017). Multi-omics analysis of serum samples demonstrates reprogramming of organ functions via systemic calcium mobilization and platelet activation in metastatic melanoma. Mol Cell Proteomics.

[65] Ratnikov B, Azablanc P, Zeev AR, Smith JW, Osterman AL, Scott DA (2015). Glutamate and asparagine cataplerosis underlie glutamine addiction in melanoma. Oncotarget.

[66] Chakraborty P, Parikh RY, Choi S, Tran D, Gooz M, Hedley ZT, Kim DS, Pytel D, Kang I, Nadig SN (2022). Carbon monoxide activates PERK-regulated autophagy to induce immunometabolic reprogramming and boost antitumor T-cell function. Cancer Res.

[67] Pathria GLJ, Hasnis E, Tandoc K, Scott DA, Verma S, Feng Y, Larue L, Sahu AD, Topisirovic I, Ruppin E, Ronai ZA (2019). Translational reprogramming marks adaptation to asparagine restriction in cancer. Nat Cell Biol.

[68] Pathria G, Verma S, Yin J, Scott DA, Ronai ZEA (2021). MAPK signaling regulates c-MYC for melanoma cell adaptation to asparagine restriction. EMBO Rep.

[69] Colombino M, Capone M, Lissia A, Cossu A, Rubino C, De Giorgi V, Massi D, Fonsatti E, Staibano S, Nappi O (2012). BRAF/NRAS mutation frequencies among primary tumors and metastases in patients with melanoma. J Clin Oncol.

[70] Iwao Y, Okamoto Y, Shirahama H, Tsukahara S, Tomida A (2021). eIF4B enhances ATF4 expression and contributes to cellular adaptation to asparagine limitation in BRAF-mutated A375 melanoma. Biochem Biophys Res Commun.

[71] Klupczynska A, Derezinski P, Dyszkiewicz W, Pawlak K, Kasprzyk M, Kokot ZJ (2016). Evaluation of serum amino acid profiles' utility in non-small cell lung cancer detection in Polish population. Lung Cancer.

[72] Xu Y, Lv F, Zhu X, Wu Y, Shen X (2016). Loss of asparagine synthetase suppresses the growth of human lung cancer cells by arresting cell cycle at G0/G1 phase. Cancer Gene Ther.

[73] Gwinn DM, Lee AG, Briones-Martin-del-Campo M, Conn CS, Simpson DR, Scott AI, Le A, Cowan TM, Ruggero D, Sweet-Cordero EA (2018). Oncogenic KRAS regulates amino acid homeostasis and asparagine biosynthesis via ATF4 and alters sensitivity to L-asparaginase. Cancer Cell.

[74] Halliday M, Radford H, Sekine Y, Moreno J, Verity N, le Quesne J, Ortori CA, Barrett DA, Fromont C, Fischer PM (2015). Partial restoration of protein synthesis rates by the small molecule ISRIB prevents neurodegeneration without pancreatic toxicity. Cell Death Dis.

[75] Albert AE, Adua SJ, Cai WL, Arnal-Estapé A, Cline GW, Liu Z, Zhao M, Cao PD, Mariappan M, Nguyen DX (2019). Adaptive protein translation by the integrated stress response maintains the proliferative and migratory capacity of lung adenocarcinoma cells. Mol Cancer Res.

[76] Zhang B, Fan J, Zhang X, Shen W, Cao Z, Yang P, Xu Z, Ju D (2016). Targeting asparagine and autophagy for pulmonary adenocarcinoma therapy. Appl Microbiol Biotechnol.

[77] Cai DJ, Zhang ZY, Bu Y, Li L, Deng YZ, Sun LQ, Hu CP, Li M (2022). Asparagine synthetase regulates lung-cancer metastasis by stabilizing the β-catenin complex and modulating mitochondrial response. Cell Death Dis.

[78] Global, regional, and national burden of colorectal cancer and its risk factors, 1990–2019: a systematic analysis for the Global Burden of Disease Study 2019. Lancet Gastroenterol Hepatol 2022, 7(7):627–647. 10.1016/S2468-1253(22)00044-9 PMC9192760 35397795

[79] Dharwadkar P, Zaki TA, Murphy CC (2022). Colorectal cancer in younger adults. Hematol Oncol Clin North Am.

[80] Schoen REPP, Weissfeld JL, Yokochi LA, Church T, Laiyemo AO, Bresalier R, Andriole GL, Buys SS, Crawford ED, Fouad MN, Isaacs C, Johnson CC, Reding DJ, O'Brien B, Carrick DM, Wright P, Riley TL, Purdue MP, Izmirlian G, Kramer BS, Miller AB, Gohagan JK, Prorok PC, Berg CD (2012). PLCO Project team: colorectal-cancer incidence and mortality with screening flexible sigmoidoscopy. N Engl J Med.

[81] Shen X, Cai Y, Lu L, Huang H, Yan H, Paty PB, Muca E, Ahuja N, Zhang Y, Johnson CH (2022). Asparagine metabolism in tumors is linked to poor survival in females with colorectal cancer: a cohort study. Metabolites.

[82] Du F, Chen J, Liu H, Cai Y, Cao T, Han W, Yi X, Qian M, Tian D, Nie Y (2019). SOX12 promotes colorectal cancer cell proliferation and metastasis by regulating asparagine synthesis. Cell Death Dis.

[83] Renaud S, Seitlinger J, Lawati YA, Guerrera F, Falcoz PE, Massard G, Ferri L, Spicer J (2019). Anatomical resections improve survival following lung metastasectomy of colorectal cancer harboring KRAS mutations. Ann Surg.

[84] Chao S, Zhang F, Yan H, Wang L, Zhang L, Wang Z, Xue R, Wang L, Wu Z, Jiang B (2023). Targeting intratumor heterogeneity suppresses colorectal cancer chemoresistance and metastasis. EMBO Rep.

[85] Guinney J, Dienstmann R, Wang X, de Reyniès A, Schlicker A, Soneson C, Marisa L, Roepman P, Nyamundanda G, Angelino P (2015). The consensus molecular subtypes of colorectal cancer. Nat Med.

[86] Toda K, Kawada K, Iwamoto M, Inamoto S, Sasazuki T, Shirasawa S, Hasegawa S, Sakai Y (2016). Metabolic alterations caused by KRAS mutations in colorectal cancer contribute to cell adaptation to glutamine depletion by upregulation of asparagine synthetase. Neoplasia.

[87] Zhang MS, Cui JD, Lee D, Yuen VW-H, Chiu DK-C, Goh CC, Cheu JW-S, Tse AP-W, Bao MH-R, Wong BPY et al: Hypoxia-induced macropinocytosis represents a metabolic route for liver cancer. Nature Communications 2022, 13(1):954. 10.1038/s41467-022-28618-9 PMC8854584 35177645

[88] Hanada K, Kawada K, Nishikawa G, Toda K, Maekawa H, Nishikawa Y, Masui H, Hirata W, Okamoto M, Kiyasu Y (2021). Dual blockade of macropinocytosis and asparagine bioavailability shows synergistic anti-tumor effects on KRAS-mutant colorectal cancer. Cancer Lett.

[89] Deng L, Yao P, Li L, Ji F, Zhao S, Xu C, Lan X, Jiang P (2020). p53-mediated control of aspartate-asparagine homeostasis dictates LKB1 activity and modulates cell survival. Nat Commun.

[90] Guardamagna I, Iaria O, Lonati L, Mentana A, Previtali A, Ugge V, Ivaldi GB, Liotta M, Tabarelli de Fatis P, Scotti C (2023). Asparagine and glutamine deprivation alters ionizing radiation response, migration and adhesion of colorectal cancer cell line. Int J Mol Sci.

[91] Escherich G, Zur Stadt U, Borkhardt A, Dilloo D, Faber J, Feuchtinger T, Imschweiler T, Jorch N, Pekrun A, Schmid I (2022). Clofarabine increases the eradication of minimal residual disease of primary B-precursor acute lymphoblastic leukemia compared to high-dose cytarabine without improvement of outcome. Results from the randomized clinical trial 08-09 of the Cooperative Acute Lymphoblastic Leukemia Study Group. Haematologica.

[92] van der Sluis I, Moricke A, Escherich G, von Stackelberg A, Holter W, Klingebiel T, Flotho C, Legien S, Tissing W, Bierings M (2013). Pediatric acute lymphoblastic leukemia: efficacy and safety of recombinant E. coli-asparaginase in infants (less than one year of age) with acute lymphoblastic leukemia. Haematologica.

[93] Silverman LB, Gelber RD, Dalton VK, Asselin BL, Barr RD, Clavell LA, Hurwitz CA, Moghrabi A, Samson Y, Schorin MA (2001). Improved outcome for children with acute lymphoblastic leukemia: results of dana-farber consortium protocol 91–01. Blood.

[94] Kloos RQH, Pieters R, Jumelet FMV, de Groot-Kruseman HA, van den Bos C, van der Sluis IM (2020). Individualized asparaginase dosing in childhood acute lymphoblastic leukemia. J Clin Oncol.

[95] Magri A, Soler MF, Lopes AM, Cilli EM, Barber PS, Pessoa A, Pereira JFB (2018). A critical analysis of L-asparaginase activity quantification methods-colorimetric methods versus high-performance liquid chromatography. Anal Bioanal Chem.

[96] Schraw JM, Woodhouse JP, Bernhardt MB, Taylor OA, Horton TM, Scheurer ME, Okcu MF, Rabin KR, Lupo PJ, Brown AL (2021). Comparison of the blood, bone marrow, and cerebrospinal fluid metabolomes in children with b-cell acute lymphoblastic leukemia. Sci Rep.

[97] Nath CE, Dallapozza L, Eslick AE, Misra A, Carr D, Earl JW (2009). An isocratic fluorescence HPLC assay for the monitoring of l-asparaginase activity and l-asparagine depletion in children receiving E. colil-asparaginase for the treatment of acute lymphoblastic leukaemia. Biomed Chromatogr.

[98] Duval M, Suciu S, Ferster A, Rialland X, Nelken B, Lutz P, Benoit Y, Robert A, Manel AM, Vilmer E (2002). Comparison of Escherichia coli-asparaginase with Erwinia-asparaginase in the treatment of childhood lymphoid malignancies: results of a randomized European organisation for research and treatment of cancer-children's leukemia group phase 3 trial. Blood.

[99] Vrooman LM, Blonquist TM, Stevenson KE, Supko JG, Hunt SK, Cronholm SM, Koch V, Kay-Green S, Athale UH, Clavell LA (2021). Efficacy and toxicity of pegaspargase and calaspargase pegol in childhood acute lymphoblastic leukemia: results of DFCI 11–001. J Clin Oncol.

[100] Maqsood B, Basit A, Khurshid M, Bashir Q (2020). Characterization of a thermostable, allosteric L-asparaginase from Anoxybacillus flavithermus. Int J Biol Macromol.

[101] Domenech C, Thomas X, Chabaud S, Baruchel A, Gueyffier F, Mazingue F, Auvrignon A, Corm S, Dombret H, Chevallier P (2011). l-asparaginase loaded red blood cells in refractory or relapsing acute lymphoblastic leukaemia in children and adults: results of the GRASPALL 2005–01 randomized trial. Br J Haematol.

[102] Russo M, Sogari A, Bardelli A (2021). Adaptive evolution: how bacteria and cancer cells survive stressful conditions and drug treatment. Cancer Discov.

[103] Vander Heiden MG, DeBerardinis RJ (2017). Understanding the Intersections between metabolism and cancer biology. Cell.

[104] Yang Q-J, Zhao J-R, Hao J, Li B, Huo Y, Han Y-L, Wan L-L, Li J, Huang J, Lu J (2018). Serum and urine metabolomics study reveals a distinct diagnostic model for cancer cachexia. J Cachexia Sarcopenia Muscle.

[105] Wu J, Li G, Li L, Li D, Dong Z, Jiang P (2021). Asparagine enhances LCK signalling to potentiate CD8(+) T-cell activation and anti-tumour responses. Nat Cell Biol.

[106] Gnanaprakasam JNR, Kushwaha B, Liu L, Chen X, Kang S, Wang T, Cassel TA, Adams CM, Higashi RM, Scott DA (2023). Asparagine restriction enhances CD8(+) T cell metabolic fitness and antitumoral functionality through an NRF2-dependent stress response. Nat Metab.

